# Remote monitoring of patients with obstructive sleep apnea syndrome treated with CPAP: a feasibility study

**DOI:** 10.3389/frsle.2025.1601389

**Published:** 2025-06-26

**Authors:** Amélie Hediger-Parolini, Florian Charbonnier, Stéphane Grandin, Hiromitsu Takahashi, Fabian Braun, Mathieu Lemay, Roger Hilfiker, Sandra Van den Broecke, Olivier Contal

**Affiliations:** ^1^School of Health Sciences Haute Ecole de Santé Vaud (HESAV), HES-SO University of Applied Sciences and Arts, Lausanne, Switzerland; ^2^Geneva Pulmonary League, Geneva, Switzerland; ^3^Swiss Center for Electronics and Microtechnology (CSEM), Neuchâtel, Switzerland; ^4^Physiotherapy Tschopp and Hilfiker, Glis, Switzerland; ^5^Service de Pneumologie et Center de Médecine du Sommeil, Hôpital Neuchâtelois, Site de Pourtalès, Neuchâtel, Switzerland

**Keywords:** obstructive sleep apnea, remote monitoring, continuous positive air pressure (CPAP), ActiGraph accelerometer, blood pressure

## Abstract

**Introduction:**

Obstructive sleep apnea syndrome (OSAS) is a prevalent sleep disorder associated with significant morbidity and mortality, particularly due to its links with cardiovascular diseases like hypertension (HT). Continuous positive airway pressure (CPAP) remains the standard treatment for OSAS, yet individualized therapy and monitoring are crucial for optimizing patient outcomes. This study explores the feasibility of utilizing connected devices to remotely monitor OSAS patients undergoing CPAP treatment.

**Methods:**

Ten patients diagnosed with OSAS were enrolled in a prospective observational feasibility study. Participants wore two wearables continuously: CenterPoint Insight Watch ™ for sleep and physical activity monitoring, and Aktiia™ bracelet for blood pressure measurement. CPAP usage data were collected using the DreamStation™ device. Data synchronization and processing were conducted using a dedicated Python script. Primary outcomes included acceptability, compliance, autonomy in device usage, and data quality. Secondary outcomes focused on the feasibility of integrating a centralized platform for analysis.

**Results:**

Acceptability among patients was reasonable, with 58% consenting to participate. However, two patients discontinued the study due to skin allergies and device interference with professional activities. Most participants demonstrated autonomy in using the devices, although two required assistance with synchronization. Data quality varied, particularly with nocturnal blood pressure measurements, affected by technical issues and individual factors. Integration of data from all devices onto a centralized platform was feasible, enabling comprehensive analysis.

**Discussion:**

The study highlighted successes in continuous remote monitoring of OSAS patients undergoing CPAP treatment. Challenges included device-related issues and manual data processing. A centralized platform for data integration and analysis proved promising for longitudinal monitoring and personalized healthcare delivery.

**Conclusion:**

This feasibility study demonstrates the potential of remote monitoring in CPAP-treated OSAS patients. Future efforts should focus on addressing technical challenges and optimizing data integration on a common platform to realize the full benefits of continuous monitoring in personalized healthcare management.

## 1 Introduction

### 1.1 Scientific background

Obstructive sleep apnea syndrome (OSAS) is a common chronic sleep disorder characterized by recurrent episodes of collapse, complete or partial, of the upper airway during sleep, leading to gas exchange impairment and frequent arousals. The main risk factors for OSAS are male sex, age, obesity and craniofacial/upper airway abnormalities (Young et al., 2004). OSAS has an estimated prevalence of 23% in women and 49% in men aged 40 years or more (Heinzer et al., 2015).

OSAS severity rating has been usually defined by apnea-hypopnea index (AHI) clinical thresholds (i.e., mild ≥ 5 to < 15 events/h, moderate ≥ 15 to < 30 events/h; and severe ≥ 30 events/h) recorded during polysomnography (PSG) or polygraphy (PG) (Pevernagie et al., 2020). However growing evidence shows that disease severity cannot be evaluated solely by the AHI (Pevernagie et al., 2020). Others physiological parameters and clinical symptoms (e.g., desaturation amplitude, event duration, pulse wave amplitude, daytime sleepiness, and other clinical manifestations) also play a role in the evaluation of the disease severity, yet precise clinical markers still need to be defined (Pevernagie et al., 2020).

OSAS is a heterogeneous disease with a wide range of clinical manifestations. Bailly et al. were able to identify eight distinct clinical phenotypes using clustering analysis which are mostly defined according to sex, age, body mass index, comorbidities, AHI, and symptoms (i.e., daytime sleepiness) (Bailly et al., 2021). This strengthens the need of individualized care with a global consideration of the different component of disease severity and not solely AHI.

Untreated, OSAS is an important cause of morbidity and mortality. Consequences include daytime sleepiness, cognitive dysfunction, reduced work performance and poor health-related quality of life (Punjabi, 2008). Moreover, OSAS has been linked to cardiovascular problems and is currently recognized as an independent risk factor for systemic hypertension and atrial fibrillation among other cardiovascular diseases (Punjabi, 2008).

The evidence of OSAS causing secondary heart diseases is becoming strong, particularly for hypertension (HT) (Wang et al., 2014). Prevalence of HT in OSAS is 50.5 %, and even up to 58.4 % in severe OSAS subgroup compared to 30.4 % in controls (Wang et al., 2014). Studies showed that OSA is associated with nocturnal blood pressure fluctuation (NBPF) and that patients are more likely to present a non-dipping BP pattern, where nocturnal normal decrease of BP is affected (Wang et al., 2014).

OSAS treatment necessitates a multidisciplinary approach and individualized therapy which varies from the disease presentation (i.e., phenotype), yet continuous positive airway pressure (CPAP) ventilation remains the standard treatment in most patients. A recent meta-analysis of randomized control trials (RCTs) demonstrated a reduction in 24-h mean BP of −2.6 mmHg with CPAP treatment (Patil et al., 2019). Nevertheless, cardiovascular benefits of CPAP treatment do not seem to apply equally to all patients and further research are needed to assess BP response on different subgroups with OSA (Patil et al., 2019).

The ANDANTE (effect of treatment for obstructive sleep apnea on blood pressure: an individual patient data meta-analysis) “project” already highlighted the desire to identify individual predictors of BP response to treatment and to provide information about the effect of OSA treatment in the less studied patients' phenotype (Pengo et al., 2021).

To achieve health benefits, CPAP therapy is directly correlated to adherence with a consensus of > 4 h use time per night (Patil et al., 2019; Weaver et al., 2007). It has been shown that there is a dose-response relationship between the use of CPAP and the objective and subjective sleepiness (Patil et al., 2019). This relationship has also been demonstrated between increased CPAP usage and a decreased healthcare resource utilization (Malhotra et al., 2023).

However, dose-response to adherence above this threshold is unclear and the relative CPAP use necessary during sleep time impacts cardiovascular health (Masa and Corral-Penafiel, 2014).

An objective measurement of the sleep time of the patients seems necessary for an individual estimation of CPAP compliance. The American Academy of Sleep Medicine has recognized actigraphy as a useful adjunct in the clinical assessment of sleep disorders (Morgenthaler, 2007).

Using home actigraphy device to estimate sleep time under CPAP treatment could provide a good index to evaluate the impact of treatment in sleep apnea (Gagnadoux et al., 2004).

Recently, new technological advances have made it possible to non-invasively monitor 24 hours BP at the wrist by photoplethysmography (PPG) (Aktiia™ bracelet, Switzerland) (Vybornova et al., 2021). A recent up to date review highlighted the advantages of the home blood pressure monitoring (HBPM) compared to the ambulatory method (ABPM) (Cepeda et al., 2023). Benefits of getting values over several consecutive days reduces the white coat effect and provides a more reliable estimation of BP, but also an average of the daily and nightly variability in BP.

Being able to measure sleep duration objectively trough actigraphy device would highlight the importance of sleep duration on the blood pressure profile. Recent observational studies showed that short sleep duration is associated with hypertension risk and reduced nocturnal dipping (Makarem et al., 2019). In addition, it also appears to be independently related with hypertension risk in OSA patients (Ren et al., 2018) Objective, longitudinal measurements of these 2 variables would allow to determine whether CPAP treatment can also contribute to lower BP in patients with OSA.

The studies conducted so far on the fluctuation of BP have evaluated the absolute parameters over 24 h and in a punctual state. Knowing the fluctuation of BP in a continuous way would allow to assess the evolution of BP variables and the potential effects of a CPAP treatment in OSAS patients. This opens new perspectives in prevention and clinical management of patients.

### 1.2 Specific outcomes: primary and secondary

#### 1.2.1 Primary outcomes

The primary objectives of this feasibility study are to assess the acceptability, compliance, and autonomy of patients in using these connected devices, as well as the quality of the signals, synchronization and transfer of data collected by the devices.

- To assess the proportion of patients who have agreed to take part in the study: acceptability.
- To assess the proportion of patients who completed the study to the end of the defined protocol: compliance.
- To assess the proportion of patients who were able to use the connected devices appropriately and autonomously (continuous wearing, charging, synchronization and calibration): autonomy.
- To assess the quality of the data synchronized and measured by the devices (% loss of data or quantity of data over the duration of the protocol).

#### 1.2.2 Secondary outcome

The secondary objective is to assess the feasibility of using all the data collected remotely from patients over 42 consecutive days. To integrate the variables measured by the connected devices on a centralized platform to establish a statistical analysis aimed at integrating artificial intelligence.

This AI process would make it possible to assess the effect of CPAP treatment on the various variables measured. In other words, to assess whether there is a dose effect between absolute use and relative use (i.e., CPAP use time/sleep time) of CPAP on blood pressure, with the aim of optimizing the individual management of OSAH patients.

## 2 Methods

### 2.1 Participants and eligibility criteria

As a prospective observational feasibility study, 10 patients were referred to the feasibility study by two primary care pulmonologists in Geneva, Switzerland. As soon as the diagnosis of OSA and the prescription of CPAP were made, eligible participants were proposed to enter the study. Patients aged at least 18 years or older with an apnoea–hypopnoea index (AHI) of more than 15 events per hour, *diagnosed by ambulatory polygraphy*, were deemed eligible. Exclusion criteria were previous exposure to treatment for OSA, language barriers, any behavioral disorder or difficulty likely to interfere with cooperation or adequate understanding of CPAP treatment, and age over 70 since the Aktiia™, device has not been validated for this population category. All included patients provided written informed consent to participate in the study. The study protocol was approved by the cantonal research ethics committee of Geneva (CCER).

### 2.2 Study design

Patients were referred by pulmonologists who prescribed the study. Upon meeting the inclusion and exclusion criteria, patients were provided with information and consent documents. Subsequently, study investigators reached out to the patients to address any questions and arrange their inclusion in the protocol. For each participant, the study lasted for 2 months from inclusion, during which they attended two clinical visits and one teleconsultation.

During the initial inclusion appointment, the following data were collected: age, gender, body mass index, and subjective daytime sleepiness measured using the Epworth Sleepiness Scale (ESS) (Johns, 1991). Participants were then equipped with two devices, namely CenterPoint Insight Watch (ActiGraph™, Pensacola, USA) and Aktiia™ bracelet (Aktiia SA, Neuchâtel, Switzerland), which they were instructed to wear throughout the protocol. The Actigraph ™ device continuously monitored sleep and wake phases, as well as physical activity. The Aktiia™ device is a cuffless monitor that measured blood pressure allowing for BP monitoring for several weeks.

Participants were given guidance on the practical use and functionality of both devices to ensure their autonomy in wearing, charging, synchronization, and Aktiia™, calibration 30 days after the bracelet's initiation. Additionally, dedicated applications for each device were installed on their mobile phones, facilitating regular data synchronization from both devices.

A baseline phase of 8 days started from the inclusion day to measure blood pressure variables (Aktiia™), daily sleep, and physical activity (Actigraph™) before CPAP treatment. A follow-up CPAP initiation appointment was scheduled 8–10 days later. This consultation took place at the Geneva Pulmonary League (LPG) and was conducted by a member of the healthcare team, and the treatment was initiated following the usual patient care protocol. All patients were treated with the CPAP device DreamStation™ (Philips Respironics, Pennsylvania, USA). A subsequent titration visit was scheduled to adjust CPAP settings based on usage feedback visible on the Care Orcherstrator™ platform.

During the study period, which lasted 42 days from inclusion, the following data were collected and remotely observed: daytime and nighttime blood pressure (BP) parameters (systolic, diastolic and heart rate), sleep, physical activity (PA), and CPAP parameters (time in use, pressure, leaks, residual respiratory events, and adherence). A teleconsultation was scheduled 30 days after the start of the study to ensure the proper functioning of devices and the successful calibration of Aktiia™.

The end of the study was scheduled 42 days later at the LPG to record post-study variables [age, gender, body mass index, subjective daytime sleepiness measured by the Epworth Sleepiness Scale (ESS)]. Participants returned the two bracelets and provided feedback to address questions regarding the feasibility of the study.

### 2.3 CPAP treatment

According to our standard practice, CPAP initiation and habituation phases typically extend over a period of 1 month and include phone appointments with a sleep care giver. During the first appointment, education about the disease and the CPAP device is provided, the adequate interface is identified, and the way to set the CPAP for titration with auto-adjusting (APAP) mode is explained. All CPAP data and settings (time in use, pressure, leaks, residual respiratory events, and adherence) are available on Care Orcherstrator™.

### 2.4 Blood pressure measurement

Blood pressure was measured by Aktiia™ Bracelet, a CE-marked blood pressure monitor worn at the wrist (Vybornova et al., 2021). It is a non-invasive monitor using PPG technology to automatically estimate BP from an optical wrist measurement throughout the day and night. To estimate BP values, Aktiia™ uses a pulse wave analysis technique following a calibration process using an oscillometric BP monitor (i.e., a BP cuff measurement). To ensure the accuracy of the BP estimate, the calibration process must be repeated every 30 days (Theiler et al., 2023; Alexandre et al., 2023). Throughout the day, regular measurements are automatically initiated, approximately once per hour, when the user is stationary to ensure optimal conditions for the PPG measurement (Sola et al., 2021). The data are then transferred via Bluetooth to the Aktiia™ mobile app to a secure cloud server. *Blood pressure measurements were considered valid if they met the signal quality criteria defined by Aktiia's proprietary algorithm. Measurements were automatically discarded if the signal quality was insufficient, for example due to excessive movement or improper sensor positioning. In addition, an initial calibration with the provided electronic blood pressure monitor was required upon first use, and then repeated monthly to ensure ongoing measurement accuracy. Daytime and nighttime periods were defined by default: the daytime period was set from 6:00 a.m. to 10:00 p.m., and the nighttime period from 10:00 p.m. to 6:00 a.m*.

### 2.5 Sleep and physical activity measurement

Objective sleep and activity parameters were recorded by a validated medical grade actigraphy monitor (The ActiGraph™ CentrePoint™ Insight watch). ActiGraph™ and the Insight watch are CE marked It captures and records high-resolution raw acceleration data, which is converted into objective activity and sleep measurements (daily energy expenditure (calories) and steps, % of moderate to vigorous physical activity intensity (MVPA), activity periods, sleep latency, total sleep time, wake up after sleep onset and sleep efficiency) using specific algorithms (Quante et al., 2018). Raw data from the monitor were automatically transmitted via Bluetooth to the CentrePoint™ Connect mobile app and then synchronized through CentrePoint™, the secure cloud-based system that provides instant access to real time patient outcomes and raw data files.

### 2.6 Data synchronization and processing

Data of all three devices (ActiGraph™, Aktiia™ and CPAP) were synchronized offline using a dedicated Python script. To do so the data were temporally aligned using the timestamps provided by each of the devices. In addition, for each of the 10 patients the sanity and completeness of the data was validated by visual inspection. For each night, total sleep time and total CPAP usage time was calculated by summing all sleep (via ActiGraph) and CPAP activity (via CPAP). In addition, diurnal BP averages were calculated by averaging Aktiia BP measurements over 1 day, for the time where the ActiGraph™ device did not indicate any sleep. Similarly, nocturnal BP averages were calculated by averaging Aktiia BP measurements between midday of the previous day and midday of the subsequent day, for the time where the ActiGraph™ device indicated that the patient was asleep.

### 2.7 Statistical analysis plan

As this was a prospective observational feasibility study, the sample size for this protocol was a convenience sample as described in the CONSORT guidelines for pilot and feasibility studies (Vybornova et al., 2021). For this protocol we included 10 patients.

The primary objective of the study was to investigate the feasibility of collecting the data obtained by the various monitoring tools. The telemonitoring platform was monitored weekly throughout the study to ensure data availability and integrity. In the event of missing or non-compliant data, the problem was immediately investigated to correct any problems.

## 3 Results

**Figure 1:**
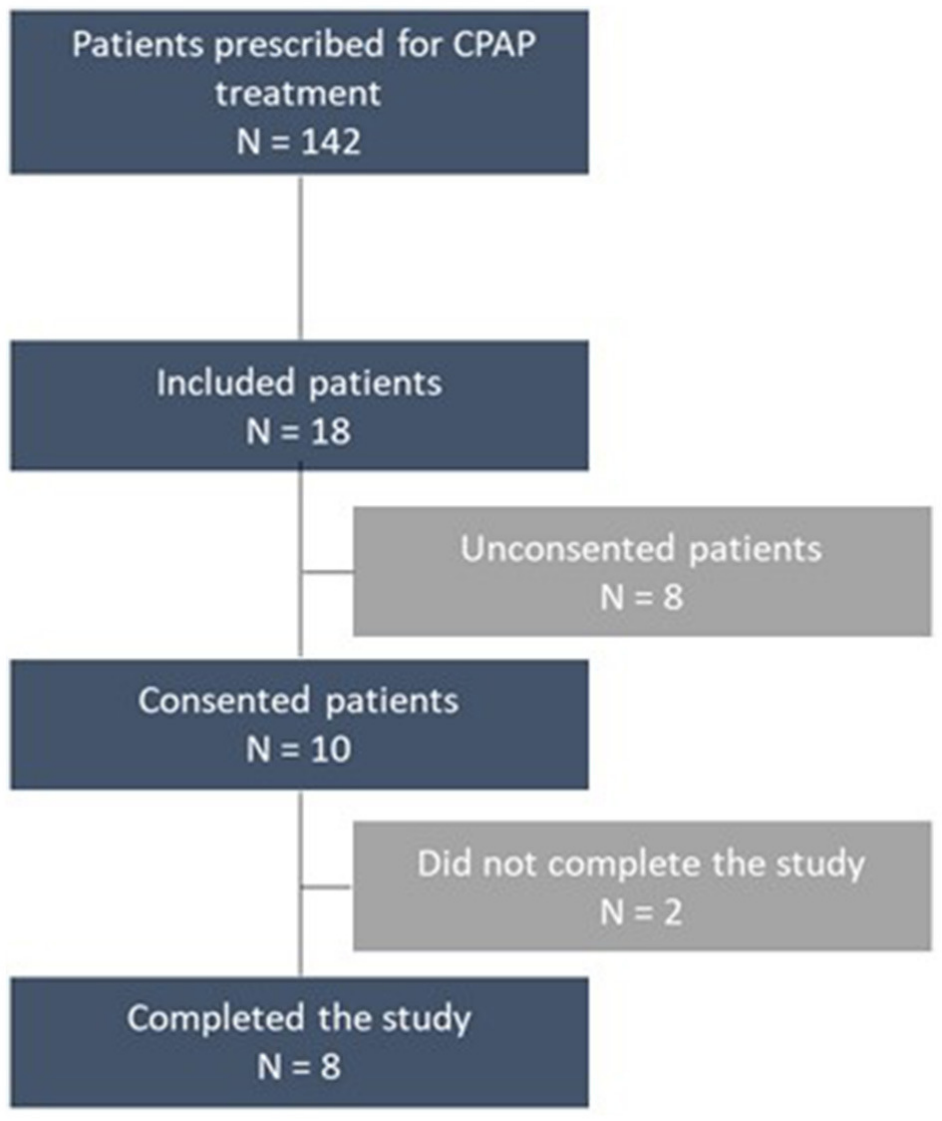
Study flowchart.

Since recruitment began in March and continued until October 2023, the LPG received 142 prescriptions from the two prescribing pneumologists. As we did not receive many consents from the specialists, the recruitment strategy was modified during the recruiting process, and we then directly and actively contacted each patient referred to the LPG to ask them for consent to participate. During this active recruitment phase, 18 patients were contacted directly by us and 10 agreed to take part in the project (Table 1). Two patients did not complete the protocol (2/10) (Figure 1).

**Table 1:** Baseline characteristics.

**Subjects (*n* = 10)**	**Median**	**IQR [first and third quartile]**
Age (years)	55	[45,25–57,5]
Males (%)	40	
BMI (kg/m2)	32.0	[29,5–38,4]
Waist circumference (cm)	115	[105,5–125,8]
Neck circumference (cm)	41	[37,8–43,8]
AHI (events/h)	29.2	[25,5–42,5]
ESS score	10.5	[7,5–11]

Results are displayed as median, interquartile range [first and third quartile]. BMI, body mass index; AHI, apnea-hypopnea index; ESS, Epworth Sleepiness Scale.

### 3.1 Primary outcomes

#### 3.1.1 Acceptability

The first criterion of this feasibility study was to assess participation consent to take part in the study. 58% of the patients (10/18) contacted agreed to participate in the study as part of their CPAP treatment (Figure 1).

#### 3.1.2 Compliance

Among the 10 participants, 2 (20%) did not complete the study. One of the reasons was due to a skin allergy to the ActiGraph™ device and the other to disturbance of the bracelet in the context of the participant's professional activities (Figure 1).

#### 3.1.3 Autonomy

Patient compliance and autonomy in the use of the connected devices were two criteria assessed in this feasibility study. The purpose of the inclusion visit was to provide patients with the information they needed to wear, synchronize, charge, and calibrate the two connected bracelets. During the protocol, a single telecommunication was planned to ensure that the usage, continuous wearing, data synchronization and calibration procedure of the Aktiia™ wristband went successfully. This intermediate contact was an opportunity to assess patient compliance and autonomy. During the final visit, feedback was given by the patients in order to evaluate these criteria at the end of the study.

Over the entire protocol, two patients (20%) were unable to use the devices independently. The punctual data synchronization via Bluetooth and the calibration requested by Aktiia™ after 30 days was carried out with assistance.

#### 3.1.4 Data accuracy and wearing devices

One of the primary objectives of this study was to assess the quality of the data measured and synchronized by the devices and their ability to be transferred successfully. This is illustrated by the number of values measured and transferred with quality, but also by the time of use and/or the percentage of data measured and/or recorded continuously.

##### 3.1.4.1 CPAP usage time

The CPAP treatment machine was started approximately 10 days after the start of the protocol. The phase of habituation and adjustment of individual settings may influence the time and quality of use of the machine. The usage data were transferred to a Care Orcherstrator™ platform, which easily indicated the time the machine was used each night and provided information on the patient's adherence to the treatment. Table 2 shows an average use for all patients of 334.3 (min/day) for the duration of the protocol. Patients wore it on most days of the protocol. Missing data concern the two subjects who did not complete the study and stopped before initiating treatment.

**Table 2:** Data wearing, accuracy and usage time—blood pressure, sleep and physical activity, CPAP.

**Subjects**	**Blood pressure: Aktiia**								**Sleep & physical activity: Actigraph**			**CPAP**	

	**Wearing nights/days/full days protocol (nb)**	**Nocturnal accuracy (%)**	**Total nocturnal data count (nb)**	**Mean nocturnal data count (nb/night)**	**Diurnal accuracy (%)**	**Total diurnal data count (nb)**	**Mean diurnal data count (nb/day)**	**Total data count (nb)**	**Mean sleep time/day, minute (SD)**	**Wearing time/day (%)**	**Wearing days (nb)**	**Mean CPAP using time/day, minute (SD)**	**Wearing days (nb)**
S01	33/32/42	75%	334	10,4 (2,7)	51%	574	17,4 (5,7)	908	453,9 (90,6)	73%	32/42	295 (135,4)	25/25
S02	36/38/38	92%	503	13,6 (4,3)	100%	970	25,5 (7,4)	1473	454,1 (72,9)	94%	41/41	370,5 (114,6)	31/34
S03	24/29/29	42%	139	4,8 (4,2)	29%	299	10,3 (4,5)	438	421,8 (93,3)	85%	41/41	359,6 (104,6)	30/30
S04	39/39/41	75%	427	10,4 (4,5)	57%	665	16,6 (8,2)	1092	515 (185,5)	81%	41/41	259,3 (64,2)	21/28
S05	6/9/12	47%	37	3,4 (3,9)	45%	135	12,3 (8,2)	172	494 (297,3)	26%	10/11	N/A	N/A
S06	28/33/41	72%	231	5,8 (4,5)	71%	444	10,8 (7,7)	675	486,2 (144,5)	80%	40/41	404,3 (68,2)	26/29
S07	8/8/11	67%	58	5,3 (4,4)	63%	127	12,7 (8,5)	185	507,4 (164,8)	86%	11/11	N/A	N/A
S08	40/39/41	61%	297	7,2 (3,2)	68%	741	18,5 (5,9)	1038	434,9 (96,8)	91%	41/41	205,3 (89,6)	28/29
S09	44/44/44	85%	530	12,0 (3,1)	67%	1106	25,1 (5,9)	1636	486 (90)	90%	44/44	376,5 (91,1)	26/29
S10	39/41/42	76%	339	8,7 (2,5)	75%	704	17,2 (4,6)	1043	445 (116,5)	93%	40/42	374,8 (75,4)	34/34
All subjects		**69%**	**2895**	9,4 (4,3)	**62%**	**8660**	**18,4 (7,7)**	**866 (498,9)**	**465,2 (130,1)**	**80%**		**334,3 (114,3)**	

Quantity of nocturnal and diurnal valid blood pressure data measured by the Aktiia percent (%) and number and wearing days (nb) over the duration of the protocol. The wearing nights/days were caclulated as follows: nbr of data at 0 means = not worn, nbr of data >0 = worn. The number of nocturnal and diurnal data obtained were transmitted via the Aktiia platform algorithm, and summed over all the days in the protocol. The % of nocturnal and diurnal valid data was calculated as follows: total nb of nocturnal data measured/number of nights in the protocol, idem for diurnal.

Sleeping time (minute) per day; wearing time (%) and days (nb) of the Actigraph device per subject, over the duration of the protocol. Sleep time was obtained directly using the Actigraph platform's algorithm/data. The number of days worn was calculated by summing the days with data (0 = not worn >0 = worn), and the % of time was obtained by relating it to the total minutes over 24 h.

Minute per day and number of days of CPAP use for each patient over the duration of the protocol; N/A, no data available. The Care Orcherstrator™ platform provided information on CPAP use timeslots (start and end times of use) over one night. The sum of total minutes of use was caclulated.

##### 3.1.4.2 Blood pressure measurement

Blood pressure parameters were measured continuously using the Aktiia™ wristband. Measurements were frequently taken when the subject was at rest and when the measurement could be made with adequate stability (Theiler et al., 2023; Sola et al., 2021). The data accuracy was then translated as “valid” or “invalid” depending on the quality of the measurement. Data quality was assessed by the number of valid measurements taken at night and during the day. The average number of valid data at night was nearly 2 times lower (*N* = 289) than during the day (*n* = 576) (Table 2). Most subjects obtained valid data greater than invalid data except for two subjects (S03 and S05). One subject (S03) out of 10 reported technical problems with the calibration of the Aktiia™ wristband. From the initial calibration, the measurement could not be performed correctly. The patient was forced to repeat the calibration many times before achieving a valid measurement. The same patient did not obtain a high percentage of valid Aktiia™ data over the entire protocol.

##### 3.1.4.3 Sleep and physical activity measurement

Both sleeping and waking phases as well as physical activity parameters (steps, MVPA) were measured by the ActiGraph™ wristband. The daily sleep time corresponds to the total sleep phases detected over 24 h. Certain phases of inactivity were detected as “sleep” and others as “not worn”, thus enabling the continuous wearing of the bracelet to be assessed. The device had to be removed whenever it met water (e.g., for care or showers). Table 2 illustrates that most subjects seemed to have worn it most of the time (80%) on average and almost every day of the protocol, except for S01 who had a holiday break in the middle of the protocol and S05 who interrupted the study because of intolerance and allergy to the strap.

### 3.2 Secondary outcome

**Figure 2:**
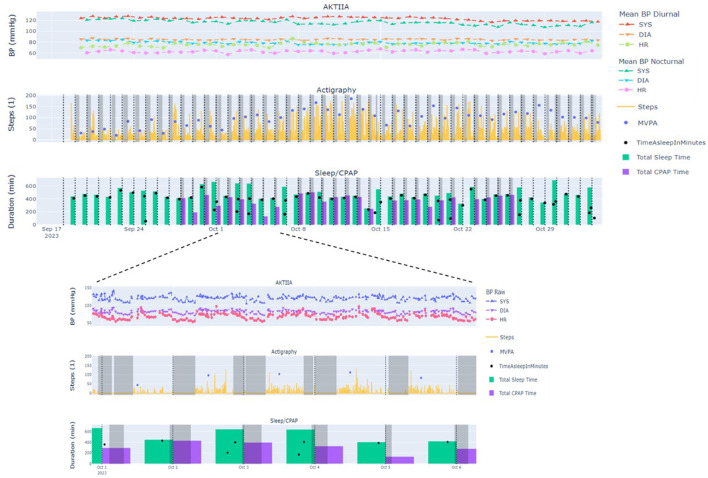
Data synchronization—overview and focus on a specific period. Overview of all data measured over the duration of the protocol for a subject and focus on data from a selected period of the protocol BP, Blood pressure (mmHg); SYS, systolic; DIA, diastolic; HR, Heart rate (bpm); MVPA, moderate to vigorous physical activity (min).

The secondary outcome of this study was to assess the feasibility of using all the data collected remotely from patients over 42 consecutive days. The aim is to be able to synchronize all the signals measured by the connected objects on a centralized platform, to establish a statistical analysis with a view to integrating artificial intelligence at later stages. The figure below (Figure 2) illustrates the visualization tool used to validate the sanity and completeness of the data (by visual inspection), by the example of data collected for one patient over the duration of the entire protocol. This figure gives an overview of the data and values measured by the connected devices on a common time base. It shows the ability to obtain raw data from all the devices simultaneously and to zoom in one selected phases of the protocol with access to the daily averages of the parameters measured continuously.

## 4 Discussion

This feasibility study formalized the collection of continuous data from patients starting CPAP treatment. For an average of 40 days, the study collected the subjects' hours of sleep and time awake, as well as mean systolic, diastolic and heart rate values for blood pressure, physical activity and CPAP usage. After the first 3 months of the protocol, the recruitment procedure had to be modified. During the first 3 months, only two patients were included. The main barrier to patient recruitment identified in this study was the lack of time available to physicians during consultations to present the research protocol. As a result, patients were not provided with the opportunity to express their interest in participation. To overcome this limitation, a revised recruitment strategy was implemented whereby prescribers referred potentially eligible patients to the research team. The investigators then directly contacted these patients to present the study protocol and collect their consent to participate. This change in recruitment then optimized inclusions, with two patients following the protocol at all times.

The majority completed the study without reporting any concerns, difficulties or discomforts that could influence their participation. The reasons for discontinuing participation in the present study were due, for one, to an allergic skin reaction to the Actigraph™ wristband, for the other, to incompatibility with daily work gestures and tasks.

During the study, the patients had no problems synchronizing and calibrating the devices (8/10. For two other patients, this procedure had to be carried out with telephone assistance.

Teleconsultation after 30 days provides support and answers any practical questions. However, patients could be offered an automatic synchronization system for all their data, to simplify the use of connected devices. The use and wearing of the different connected devices enabled all the data to be collected and transferred to their respective platforms. Regarding CPAP usage data, the Care Orcherstrator™ platform did not allow raw data from the CPAP device to be exported. The use of raw data should be developed to avoid manual retranscription. Other brands of devices should allow this transfer of raw data as in the protocol of Pontier-Marchandise et al. (2023).

The Aktiia wristband is a new-generation device and has not presented any problems with connection or data collection. Today, the main problem with this wristband remains the reliability of the measurements. A recent article compared cuffless wearable BP Aktiia™ device with conventional out-of-office methods, such as ambulatory (ABPM) and home BP monitoring (HBPM). Daytime measurements are comparable, while nighttime measurements currently appear to be somewhat less adequate compared to conventional cuff-based ABPM (Tan et al., 2023). However, Aktiia™ device has the advantage of providing a more complete BP profile with reliable and comparable daytime BP data (Almeida et al., 2023). Although nocturnal data provide lower absolute reliability, they do allow longitudinal monitoring of values for individual patients.

The Aktiia™ wristband provided a large number of measurements over the entire protocol, some subjects did not obtain a high percentage of valid measurements, especially at night. These differences in quality of measurement again appear to be inter-individual. It is nevertheless conceivable to use this tool to assess the effects within the same patient. According to Vybornova et al. (2021), “the quality of the optical signals recorded by Aktiia™ Bracelet would compromise the reliability of the pulse wave analysis algorithms in patients with darker skin pigmentation (due to the increased absorption of the light), as well as in patients with increased hair follicle density [due to the introduction of an optical barrier” (Vybornova et al., 2021)].

Concerning the Actigraph™ wristband, the data could be easily synchronized and analyzed. The bracelet provided all physical activity (MVPA, steps), wake and sleep data. However, sleep periods needed to be regularly readjusted and identified manually. Despite the algorithm used to detect sleep/wake phases (Kim et al., 2013), some sleep or wake phases were more difficult to objectify. Offering occasional remote monitoring (at 10-day intervals) with a questionnaire seems to be a solution that could help to understand certain specific periods identified as data loss, non-use of connected devices, diurnal sleep or disturbed nights, or to record certain extraordinary events (illness, naps, holidays, extraordinary activities outside the daily routine).

Wearing and use of the connected tools was done appropriately for most of the subjects over the duration of the protocol. Punctual data synchronization (for Aktiia™ and Actigraph™) was done correctly but still manually and actively by the patients. The inclusion phase of the patients and the telemonitoring conducted 30 days after the start of the protocol enabled the patients to be autonomous and compliant for most of them. Subjects who completed the study showed a high level of adherence and tolerance to CPAP treatment and connected bracelets, having worn it on the most days of the protocol. Data synchronization and processing was carried out reliably and automatically using a dedicated Python script.

As part of the initial management of patients newly diagnosed with obstructive sleep apnea (OSA) undergoing continuous positive airway pressure (CPAP) treatment, the relevance of systematic telemonitoring deserves careful consideration. The recently conducted feasibility study highlights the significant potential of continuous monitoring through connected devices to optimize treatment adherence and personalize care, particularly through the monitoring of parameters such as blood pressure, physical activity, and real-time CPAP usage. Although the majority of patients demonstrated satisfactory autonomy in using these devices, technical challenges—such as synchronization difficulties and variability in data quality—indicate the need to refine these tools for broader implementation.

Furthermore, the recent review by Pépin et al. (2025), emphasizes the importance of a multidisciplinary and personalized approach to improve long-term therapeutic adherence and the management of comorbidities associated with OSA. Telemonitoring appears particularly promising for the early identification of patients at high risk of poor adherence or cardiovascular complications, thereby enabling rapid and targeted intervention. Additionally, integrating these data into centralized platforms could facilitate comprehensive and coherent management, while optimizing the use of healthcare resources based on individual risk profiles.

However, the systematic deployment of such monitoring requires careful consideration of associated costs, acceptability by patients and healthcare professionals, and reimbursement modalities. The expected benefits—particularly in terms of reducing cardiovascular events and improving quality of life—must be carefully weighed against economic and logistical constraints. Thus, although promising, remote monitoring should perhaps initially be reserved for high-risk patient profiles or those showing poor initial adherence to CPAP before being more broadly extended to all newly diagnosed patients.

As alternatives to the devices used in the feasibility study, other medically certified connected tools may also be considered. These include the Oura Ring Gen3, which has shown reasonable agreement with polysomnography for sleep staging and nocturnal heart rate variability monitoring in validation studies (Svensson et al., 2024). A recent review (Birrer et al., 2024), provides a comprehensive analysis of current and emerging wearable technologies for sleep monitoring and intervention. This review highlights not only the validation status of several devices, including the Oura Ring, but also discusses their clinical utility, generalizability, and the challenges related to standardization and integration into care pathways. These insights support the inclusion of such wearables in future telemonitoring strategies aimed at improving OSA management through accessible, scalable, and patient-centered approaches.

This feasibility study allows us to determine the sample size for a future prospective project. We will therefore use this estimate: Given the complex structure of the data and the primary analyses (repeated measure mixed model with a random slope), there is no formula to calculate the required sample size. We simulated data based on previous own and published studies to calculate the statistical power for a set of sample sizes. We produced 1,000 random samples for each sample size. For a significance level (type-I error rate) of 5% and a power of 85% (type-II error of 15%) we would need 110 participants. To allow a dropout rate of 10% we need to include 123 participants. This means approximately 15% of all newly equipped patients would need to fulfill the participation criteria and accept to participate to reach the adequate sample over a 2-year recruitment period.

## 5 Limitations

Data could be exported easily from both the Aktiia™ and Actigraph™ wristbands, however the CPAP device does not allow the raw data to be exported. The same applies to the synchronization of the CPAP time with the timestamp. Time synchronization is done automatically for the two bracelets but must be done manually with the CPAP and the timestamp. A centralized platform would enable all the data to be synchronized and exported, with longitudinal monitoring over a single, common timestamp. Using a single connected bracelet would also optimize the feasibility of the study (adherence, compliance and autonomy). Finally, the algorithm used to detect sleep and wake phases on the ActiGraph™ revealed a lack of robustness and could be optimized.

## 6 Conclusion

In conclusion, the feasibility study successfully demonstrated the potential of using connected devices to monitor patients undergoing CPAP treatment for obstructive sleep apnea syndrome (OSAS). Despite encountering some challenges, the majority of participants completed the study, highlighting the acceptability and compliance of the approach. The study showed promising results in terms of data synchronization and processing, paving the way for future research integrating common platform for all connected devices and artificial intelligence to optimize individual management of OSAS patients. However, improvements are needed in the reliability of blood pressure measurements, particularly during nighttime, and the implementation of automated data synchronization systems to enhance user experience. Overall, this study underscores the value of remote monitoring in improving the management of OSAS and warrants further investigation with larger sample sizes and longer follow-up periods.

## Data Availability

The raw data supporting the conclusions of this article will be made available by the authors, without undue reservation.
